# The Comparative Experimental Study of Multilabel Classification for Diagnosis Assistant Based on Chinese Obstetric EMRs

**DOI:** 10.1155/2018/7273451

**Published:** 2018-02-05

**Authors:** Kunli Zhang, Hongchao Ma, Yueshu Zhao, Hongying Zan, Lei Zhuang

**Affiliations:** ^1^Information Engineering School, Zhengzhou University, Zhengzhou, Henan 450000, China; ^2^Industrial Technology Research, Zhengzhou University, Zhengzhou, Henan 450000, China; ^3^The Third Affiliated Hospital of Zhengzhou University, Zhengzhou, Henan 450052, China

## Abstract

Obstetric electronic medical records (EMRs) contain massive amounts of medical data and health information. The information extraction and diagnosis assistants of obstetric EMRs are of great significance in improving the fertility level of the population. The admitting diagnosis in the first course record of the EMR is reasoned from various sources, such as chief complaints, auxiliary examinations, and physical examinations. This paper treats the diagnosis assistant as a multilabel classification task based on the analyses of obstetric EMRs. The latent Dirichlet allocation (LDA) topic and the word vector are used as features and the four multilabel classification methods, BP-MLL (backpropagation multilabel learning), RAkEL (RAndom k labELsets), MLkNN (multilabel k-nearest neighbor), and CC (chain classifier), are utilized to build the diagnosis assistant models. Experimental results conducted on real cases show that the BP-MLL achieves the best performance with an average precision up to 0.7413 ± 0.0100 when the number of label sets and the word dimensions are 71 and 100, respectively. The result of the diagnosis assistant can be introduced as a supplementary learning method for medical students. Additionally, the method can be used not only for obstetric EMRs but also for other medical records.

## 1. Introduction

Since family planning was issued as one of the fundamental state policies in China, late marriage and late childbirth have indeed benefited the country. However, it has also led to the increasing proportion of older pregnant women especially those who are over 35 years old. The problem is exacerbated with the implementation of the *Universal Two-child Policy* in 2016. Later pregnancies are associated with higher risks of fetal abnormality and other complications, which are challenges for obstetricians [1]. Since the National Health and Family Planning Medical Affairs Commission issued the *Basic Norms of Electronic Medical Records* (Trial) [2] in 2010, medical institutions have accumulated many obstetric EMRs (electronic medical records). EMR data are big data in the medical field. They contain medical data and a large amount of patients' health information. Currently, one urgent task is how to achieve clinical information decision support with these resources in order to improve clinical treatments.

EMRs are the detailed records of medical activities written by the medical staff, in which free text (semistructured or unstructured) is one of the most important forms [3]. Using natural language processing technology to structure EMRs and extract information is a crucial step to ensure that the best possible information is contained in the EMRs. As artificial intelligence develops, automatic medical diagnosis becomes possible. In EMRs, the first course record is stored in a textual format and includes the chief complaints, physical examinations, auxiliary examinations, and other information, which can provide the foundation for admitting diagnosis. Generally, admitting diagnosis in obstetric EMRs includes more than one single diagnosis but includes normal obstetric diagnosis, medical diagnosis, and complications. The problem can be transformed into a multilabel classification task in machine learning, in which the different diagnoses can be regarded as the variable labels.

Based on the analysis of the structure and content of Chinese obstetric EMRs, the first course records are cleaned and structured in this paper. The collected Chinese obstetric EMRs are divided into complaints, physical examinations, obstetrical examinations, and auxiliary examinations. Then, the latent Dirichlet allocation (LDA) topic model is utilized to extract the features. The word vectors trained by the Skip-gram model are regarded as the features. Several multilabel classification methods are employed to diagnose the obstetric EMRs, which is an initial attempt for a diagnosis assistant based on Chinese obstetric EMRs.

## 2. Related Works

Each instance belongs to only one label in both the conventional binary class task and multiclass task, while each instance can belong to more labels in the multilabel classification. For example, the diagnosis from a doctor for one patient is usually a variety of mixed results rather than a single one. Multilabel classification has often been applied in the fields of text classification [4–6], emotional classification [7, 8], image and video classification [9–11], bioinformatics [12–15], and medical classification [16–20]. Recently, there were three research works which focus on multilabel learning (MLL). The first one improves or proposes new classification or sorting models. Zhang et al. [21] changed the original error function and proposed the BP-MLL (backpropagation multilabel learning) method on the basis of the traditional multilayer feed-forward neural networks. Li et al. [22] improved the classifier chain (CC) method and named it the ordered classifier chain (OCC). It can effectively utilize the dependency relationship among different labels. The second focus improves or proposes new feature selection models. Duan et al. [23] defined the lower approximation and dependency and designed a neighborhood rough set based on a feature selection algorithm for multilabel classification. The third focus applies MLL to new areas. Liu et al. [24] applied an MLL to choose symptoms from a Chinese coronary heart disease dataset.

In the field of medical research [16–20], Shao et al. [16] proposed an algorithm called hybrid optimization-based multilabel (HOML) to select features. HOML combined the relatively strong global optimization ability of the simulated annealing algorithm, the genetic algorithm, and the strong local optimization capability of greedy algorithm. They adopted the multilabel classifier to model coronary heart disease in traditional Chinese medicine (TCM), which significantly improved the performance. Zhang et al. [18] used multilabel learning by exploiting label dependency (LEAD) subsequently to the tongue image classification in TCM. Xu et al. [19] combined the random forest algorithm and the MLL algorithm. They then used it to select symptoms of excess chronic gastritis and establish classification models. Goldstein et al. [25], using data from I2B2 of 2008, trained one specialist classifier per class and classified obesity and its comorbidities using the MLL method. The previous research was mainly conducted on normalized public dataset or real records that included a relatively small number of labels.

In the field of diagnosis assistants, Jiang et al. [26] presented a novel computational model for the aided diagnosis of subhealth. The dataset was divided into the training set and the test set. Based on the rough set and fuzzy mathematics, the training set was used to extract important features and generated fuzzy weight matrixes. Then, the features and fuzzy weight matrixes were used to assist the diagnosis of subhealth. Tiwari et al. [27] presented the LTEM-PCA-ANN (LAW texture energy measures (LTEM), principle component analyses (PCA), and artificial neural network (ANN)) approach which can improve results with an overall accuracy of 93.34%. Then, the computational model was used to design an adequate computer-aided diagnosis (CAD) system for the classification of brain tumors to assist inexperience radiologists in the diagnosis process. Jiang et al. [28] proposed a three-layer knowledge-based model (disease-symptom-property) to diagnose a disease, which significantly reduces the dependencies between attributes and improves the accuracy of predictions.

However, very few studies have been conducted on the diagnosis assistant of the complicated Chinese obstetric EMRs up to now. Chinese is a logographic language and the Chinese EMRs are free narrative texts, which will bring challenges to a diagnosis assistant. Furthermore, the obstetrical diagnosis types are complicated, and some of their features are not easy to directly extract, which also makes it more difficult to conduct the research on a diagnosis assistant for the complicated obstetrics EMRs. In this paper, the LDA topic model and Skip-gram model are used to carry out feature selection. The methods of BP-MLL [21], RAkEL (RAndom k labELsets) [29], MLkNN (multilabel k-nearest neighbor) [30], and CC [31] multilabel classification are employed to study the automatic diagnosis of obstetric EMRs.

## 3. Materials and Data Preprocessing

### 3.1. Materials

**Figure 1:**
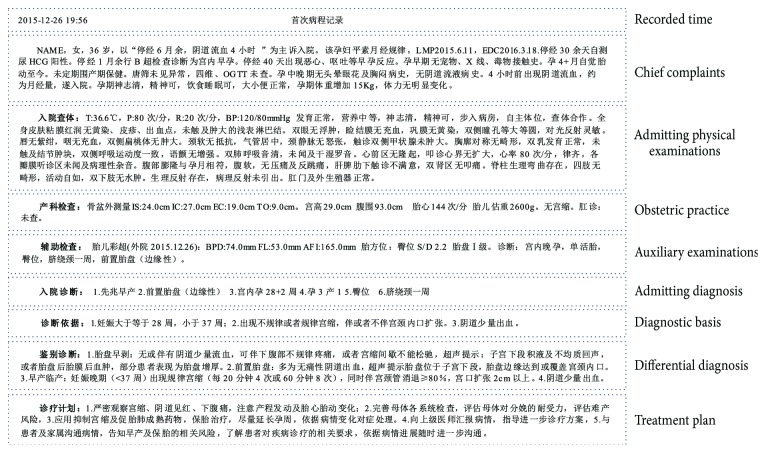
The example of the first course of disease record.

This paper takes more than 10,000 copies of Chinese obstetric EMRs as a research dataset. These data were randomly selected from 15 hospitals. Under the guidance of the *Basic Specification of Electronic Medical Records* (trial) [2], the written forms of EMRs in different hospitals vary slightly according to the actual situations in China. Charts and free text are the major forms of EMRs, and the unstructured free text is one of the main information extraction research objects. The obstetric EMR mainly includes the two parts, the course records and the discharge summary. In addition, there will be preoperative summaries, operation records and postoperative course records if a surgery is performed, and there will be newborn case records if a baby was born. In general, one course record includes one first course record, one or more daily course records (also known as ward-round records), superior doctors' ward-round records, and one discharge summary. We focus on analyzing the content and characteristics of the first course records. The first course record usually includes the recorded time, chief complaints, admitting physical examinations, obstetric practice, auxiliary examinations, admitting diagnosis, diagnostic basis, differential diagnosis, and treatment plan. An example of the first course record is shown in Figure 1.

In the first course record, the admitting diagnosis is made by the obstetricians who comprehensively analyze the patient's conditions. As is shown in Figure 1, the admitting diagnosis “宫内孕 28+2 周 (*intrauterine pregnancy* 28 +2 weeks)” can be calculated from the date of the last menstrual period in chief complaints or obtained directly from the result of auxiliary examinations, and the diagnosis “孕 3 产1 (pregnancy 3, production 1)” can be extracted from the chief complaints in the admitting records. The rest of the four diagnoses can be inferred from the features contained in the chief complaints or the previous examinations. Therefore, the admitting diagnosis in the first course record can be regarded as a multilabel classification according to the explicit or implicit features contained in the complaints or examinations.

### 3.2. Data Preprocessing

Since the collected EMRs are real cases, it is necessary to protect patients' privacy and it is inevitable that they contain some noisy data. Deidentification and data cleansing are the necessary steps for the processing of EMRs. In the process of analyzing the extracted records, the private information, such as mentions of patients, hospitals, doctors, patient's ID, location, and phone number, have all been removed from the records. Then, the essential preprocessing of the EMR data is conducted, including data cleansing, data structuration, word segmentation, and data standardization, which are described below.

#### 3.2.1. Data Cleansing

There are problems such as redundancy, missing information, and disordering due to deficiencies in the existing HIS (hospital information system). For redundant records, the records are filtered through automatically string matching. In particular, when more than one first course record is detected in one EMR, the correct one will be chosen according to the integrity of information and record time, and the others will be removed. For a missing first course record, the EMR will be deleted from the dataset. For temporal disordering, an algorithm is designed to detect the temporal error records according to the temporal logic of the obstetric treatment, and the records that include temporal errors are also removed from the dataset. Finally, the dataset contains 11,303 copies of first course records.

#### 3.2.2. Data Structuration

All content in one original EMR text is mixed together. To facilitate data analysis, the first course records are formatted in accordance with the chief complaints, admitting physical examinations, obstetric practice, auxiliary examinations, admitting diagnosis, diagnostic basis, differential diagnosis, and treatment plan, which form the experimental dataset in this paper. The record in Figure 1 is arranged according to the section of content after structuring.

#### 3.2.3. Word Segmentation

In this paper, chief complaints, physical examinations, obstetric examinations, and auxiliary examinations are used to predict the admitting diagnosis. The admitting diagnosis and the other parts extracted from the EMRs have been cleaned and structured by using the aforementioned methods, from the experimental dataset. We regard the first four parts as features and regard the admitting diagnosis as labels. The word segmentation tool ICTCLAS (Institute of Computing Technology, Chinese Lexical Analysis System) (https://codeload.github.com/NLPIR-team/NLPIR-ICTCLAS/zip/master) is put to use to segment the word in the dataset. Medical terminology and drug names obtained from the Internet and literature [32] are added to the ICTCLAS dictionary in order to improve the segmentation accuracy.

#### 3.2.4. Data Standardization

The diagnoses such as *pregnancy X + Y weeks* and *pregnancy Z production U* are the results of a calculation or complaint, so they will not be accepted as class labels. The rest of the diagnoses are accepted as class labels in the multilabel classification and form label set *L_1_* that includes 737 labels. Through the analysis of the class label set, it is found that there is more than one written form for the same category since the EMRs are extracted from different medical institutes and the doctors have personalized writing habits. For example, in set *L*_1_, “胎盘前置状态 (state of placenta previa)” and “前置胎盘 (placenta previa)” are different writing forms, but they are the same diagnosis. In this case, based on the naming rules of ICD10 (International Classification of Diseases 10) disease, after the segmentation of the diagnosis results, the similarity of labels is calculated based on the semantic method (https://my.oschina.net/twosnail/blog/370744#comment-list). The similarity *S_s_* is defined as follows:

(1) $$\begin{array}{c} S_{s}=\frac{S_{1}\times S_{2}}{\left\|S_{1}\right\|\times \left\|S_{2}\right\|}, \end{array}$$

where *S*_1_ and *S*_2_ are the semantic vector representations of the two diagnosis labels.

**Figure 2:**
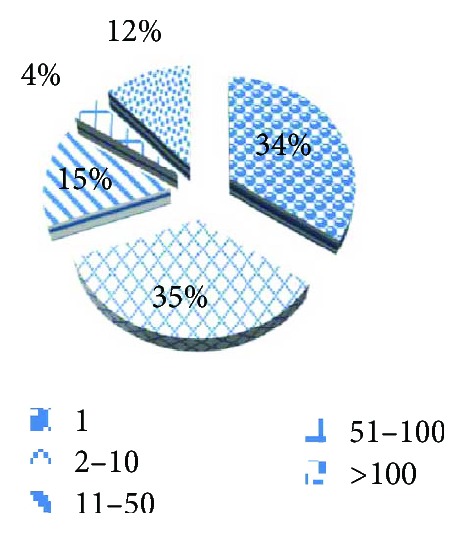
The frequency distribution of diagnoses.

Depending on the similarity calculation result, medical professionals standardize the class labels and merge the labels that have the same diagnostic results but different expressions. Finally, we get the label set *L*_2_ that contains 233 class labels. The frequency statistics are shown in Figure 2.

The number of diagnosis labels that appear once is 80, which accounts for 34% of the total. The number of diagnosis labels that appear in 2–10 is 82, which accounts for 35% of the total. The total frequency of diagnosis labels is 26,772 in the dataset. The minimum number of diagnosis labels in one instance is 1, while the maximum is 8.The average number of labels in one instance is 2.67.

## 4. Method

**Figure 3:**
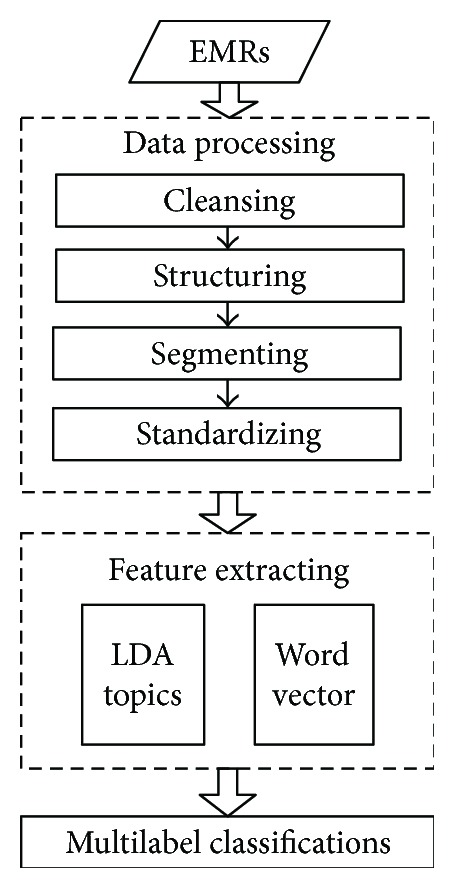
The workflow of the diagnosis assistant.

Figure 3 is the workflow of the diagnosis assistant process. Data processing has been described in Section 3.2. Feature extraction and the multilabel classification are as follows.

### 4.1. Feature Extracting

The most important stage in MML, and any classification problem, is the feature extraction in which the data are represented in a low dimensional space by the most descriptive features that maximize and characterize the interclass differences. From Figure 1, we see that there are many numerical data in EMRs, but the main written form is still free narrative text. In this paper, we utilize two methods, the LDA and Skip-gram models, to obtain features. The three-layer structure of the LDA can effectively extract the textual features of narrative texts, and Skip-gram is an efficient method for learning high-quality distributed vector representations that capture a large number of precise syntactic and semantic word relationships.

#### 4.1.1. LDA

The LDA was proposed by Blei et al. [33]. It is a three-layer Bayesian model, which has been widely applied to feature extraction. The input of the LDA model is a segmented document set *D*, and the output is the probability distribution for each document *d* under each topic *k*.

Each document *d* can be seen as an *N*-word composition and a *k*-topic composition, and the word is the basic unit in the topic. For document *d*, we choose a topic *k* from the document topic distribution *θ*, and then select a word *w* from the corresponding subdistribution *φ* in the topic *k*. It can form a document containing *N* words by repeating the above steps that are shown as follows:

(2) $$\begin{array}{c} p\left(\theta ,z,w\;|\;\alpha ,\beta \right)=p\left(\theta \;|\;\alpha \right)\overset{N}{\underset{n=1}{\prod }}\left(z_{n}\;|\;\theta \right)p\left(w_{n}\;|\;z_{n},\beta \right). \end{array}$$

The document topic distribution

(3) $$\begin{array}{c} p\left(k\;|\;d\right)=\frac{C_{dk}+\alpha }{\sum_{k=1}^{K}C_{dk}+K\alpha } \end{array}$$

and the word subject distribution

(4) $$\begin{array}{c} p\left(k\;|\;w\right)=\frac{C_{wk}+\beta }{\sum_{k=1}^{K}C_{wk}+K\beta } \end{array}$$

can be obtained by LDA, where *C*_*wk*_ is the number of times the word *w* is given the subject *k*, and *C*_*dk*_ is the number of times the document *d* is given the subject *k*.

#### 4.1.2. Word Vector

Distributed representations of words in a vector space help learning algorithm achieve better performance in natural language processing tasks by grouping similar words.Word2vec is an implementation of the model proposed by Mikolov et al. [34] that can be used to quickly and effectively express words as word vectors. It contains two kinds of training models, which are the CBOW (continuous bag-of-words) model and the Skip-gram model [35]. There are three layers, including the input layer, projection layer, and output layer. In this paper, we use the Skip-gram model to obtain the features. The CBOW model generates word vectors by using the contextual information to predict the current word. Meanwhile, the Skip-gram model generates word vectors in the opposite way by generating word vectors that utilize the current word vector to predict the word vector of possible context. In this paper, we choose the Skip-gram model to train the word vector. For the skip model, the training goal of the Skip-gram model is to maximize the value:

(5) $$\begin{array}{c} \frac{1}{T}\overset{T}{\underset{t=1}{\sum }}\sum_{-c\leq j\leq c,j\neq 0}\log p\left(w_{t+j}\;|\;w_{t}\right), \end{array}$$

where *c* is the size of the training context, and *T* is the size of the training text. The basic Skip-gram model calculates the conditional probability:

(6) $$\begin{array}{c} p\left(w_{O}\;|\;w_{I}\right)=\frac{\exp\left({v_{w_{O}}^{\prime }}^{T}v_{w_{I}}\right)}{\sum_{w=1}^{W}\exp\left({v_{w}^{\prime }}^{T}v_{w_{I}}\right)}, \end{array}$$

where **v**_*w*_ and **v**_*w*_′ are the input and the output vector representations of *w*, respectively, and *W* is the number of words in the vocabulary.

After the word vector is obtained through the Skip-gram model, the document vector can be calculated by averaging the vectors of the words contained in the document.

### 4.2. Multilabel Classification

In the training set *f*(*x*_1_, *Y*_1_), (*x*_2_, *Y*_2_),…, (*x*_*m*_, *Y*_*m*_), each instance x*_i_* is a *d*-dimensional feature and *Y*_i_ ⊆ *y* is the set of labels associated with this instance. The original error function of the traditional multilayer feed-forward neural networks is defined as follows:

(7) $$\begin{array}{c} E=\overset{m}{\underset{i=1}{\sum }}E_{i}=\overset{m}{\underset{i=1}{\sum }}\overset{Q}{\underset{j=1}{\sum }}{\left(c_{j}^{i}-d_{j}^{i}\right)}^{2}, \end{array}$$

where *E_i_* is the error of the network on *x_i_*, *c*_*j*_^*i*^ = *c*_*j*_(*x*_*i*_) is the actual output of the network on *x_i_* on the *j*th class, and *d*_*j*_^*i*^ is the desired output of *x_i_* on the *j*th class. In (7), it is assumed that each class label is independent and the relationships between labels are not considered. Zhang et al. [21] changed the original error function and changed the traditional multilayer feed-forward neural networks to the BP-MLL. The new error function is shown as follows:

(8) $$\begin{array}{c} E=\overset{m}{\underset{i=1}{\sum }}E_{i}=\overset{m}{\underset{i=1}{\sum }}\frac{1}{\left|Y_{i}\right|\left|\overset{¯}{Y_{i}}\right|}\overset{m}{\underset{\left(k,l\right)\in Y_{i}\times \overset{¯}{Y_{i}}}{\sum }}\exp\left(-\left(c_{k}^{i}-d_{l}^{i}\right)\right), \end{array}$$

where $\overset{¯}{Y_{i}}$ is the complementary set of *Y*_*i*_ in *y* and |·| measures the cardinality of a set. Specifically, *c*_*k*_^*i*^ − *c*_*l*_^*i*^ measures the difference between the outputs of the network on one label belonging to *x*_*i*_ (*k* ∈ *Y*_*i*_) and one label not belonging to it ($l\in \overset{¯}{Y_{i}}$) [21]. Therefore, the minimization of (8) will lead the system to output larger values for labels belonging to the training instance and smaller values for those not belonging to it.

## 5. Experiments

### 5.1. Experimental Design and Evaluation

In this paper, the LDA model and Skip-gram model are employed to select features. From Section 4.1, the document topic model distribution acquired from the LDA model and the word vector obtained from the Skip-gram model are regarded as the features of multilabel classification. The selected BP-MLL is compared to the RAkEL, MLkNN, and CC classification algorithms, and the effects of three factors on the experimental results are, respectively, considered.

First, as shown in Figure 2, the frequency of diagnostic labels has an uneven distribution, and the proportion of low-frequency labels is high. Therefore, the experiments are performed on different frequency label sets. Second, the LDA is used to extract features, and the number of different features has an impact on the experimental results. Therefore, LDAs with different topics are investigated. Third, the number of the word vector dimensions in the Skip-gram also influences the experimental results. Therefore, experiments with different word dimensions are also conducted.

There are three groups of experiments in this section. In the first group, the topic number of the LDA is set to 120, and the word vector dimension is set to 100. The experiments are conducted to compare the classification performance of the different numbers of the label set. In the second and the third treatments, the size of the diagnostic label set remains 71. The second group of experiments compares the results of different topics in the LDA method, and the third compares the results of various numbers of vector dimensions in the Skip-gram model.

Hamming loss, one-error, coverage, ranking loss, and average precision are used as evaluation indicators. Hamming loss (HL) is defined as follows:

(9) $$\begin{array}{c} {hloss}_{s}\left(h\right)=\frac{1}{p}\overset{p}{\underset{i=1}{\sum }}\frac{1}{Q}\left|h\left(x_{i}\right)∆Y_{i}\right|. \end{array}$$

It evaluates the error rate between the real mark of the instance and the resulting mark of the system. It is that the instance has the possibility of marking *Y_i_* but not being identified or not having the token *Y_i_* being misjudged. A smaller HL indicates a better classification effect.

One-error (OE) is defined as follows:

(10) $$\begin{array}{c} one‐error\left(f\right)=\frac{1}{p}\overset{p}{\underset{i=1}{\sum }}\left[\arg\underset{y\in Y}{\max}f\left(x_{i},y\right)\notin Y_{i}\right]. \end{array}$$

It evaluates the likelihood that the highest ranked marker is not the true markup of the instance in the category sorting sequence of the sample. In single label learning, it evolves into a general classification error rate. A smaller OE indicates a better classification effect.

Coverage (C) is defined as follows:

(11) $$\begin{array}{c} coverage\left(f\right)=\frac{1}{p}\overset{p}{\underset{i=1}{\sum }}\underset{y\in Yi}{\max}\mathrm{rank}f\left(x_{i},y\right)-1. \end{array}$$

It evaluates the average number of search depths in the category sorting sequence of the instance to cover proper labels of the instance. A smaller C indicates a better classification effect.

Ranking loss (RL) is defined as follows:

(12) $$\begin{array}{c} {rloss}_{s}\left(f\right)=\frac{1}{p}\overset{p}{\underset{i=1}{\sum }}\frac{1}{\left|Y_{i}\right|\;|\;\overset{¯}{Y_{i}}\;|\;}\left|\left\{f\left(x_{i},y_{1}\right)\leq f\left(x_{i},y_{2}\right),\left(y_{1},y_{2}\right)\in Y_{i}\times \overset{¯}{Y_{1}}\right\}\right|. \end{array}$$

It evaluates the likelihood of a sorting error in the category sort sequence of the sample. It is likely that the sample has a mark on it that is lower than the ranking of the marker that it does not have. A smaller RL indicates a better classification effect. Average precision (AP) is defined as follows:

(13) $$\begin{array}{c} {avgprec}_{s}\left(f\right)=\frac{1}{p}\overset{p}{\underset{i=1}{\sum }}\frac{1}{\left|Y_{i}\right|}\sum_{y\in Y_{i}}\frac{\left|\left\{{\mathrm{rank}}_{f}\left(x_{i},y^{\prime }\right)\leq {\mathrm{rank}}_{f}\left(x_{i},y\right),y^{\prime }\in Y_{i}\right\}\right|}{{\mathrm{rank}}_{f}\left(x_{i},y\right)}. \end{array}$$

It evaluates the case where the marker with a large membership value is still an associated mark in the category sort queue of the sample. It reflects the average accuracy of the predictor class. A higher AP indicates a better classification effect.

### 5.2. Experimental Results on the Different Sizes of the Label Set

In this group of experiments, LDA topic number *K* is set as 120 and the word vector dimension **T** is set as 100. First, the size of label set *L*_2_ is set as 233. It includes all class labels in the data set. The results are shown in Table 1. In the table, for each criterion, “↓” indicates “the smaller the better,” while the “↑” indicates “the bigger the better.” It can be seen that in all indicators, the experiments using word vector feature obtain the best results. MLkNN is the best result in HL, OE, and AP indicator. BP-MLL presents the best results for RL and C. Moreover, BP-MLL also ranks second in terms of the other three indicators.

As seen from Table 1, MLkNN using word vector feature obtains the best result, but its AP is only 0.7272 ± 0.0081. According to the results shown in Figure 2, there are 80 diagnostic labels whose frequency is only 1, and 82 diagnostic labels whose frequency is between 2 and 10. This adds up to a total of 162. The analysis of these labels reveals that there are three different situations. First, as the EMRs have not been classified, the labels are taken in all obstetric hospitalization of patients. Some obstetric diagnoses are atypical, such as obesity, allergic dermatitis, and others. Second, because of different writing habits, some doctors may write the diagnosis, such as “single pregnancy,” which may rarely be written in the normal record by most doctors. Third, some of the results are relatively rare, such as “fetal nasal bone loss.”

These labels appear only once in a data set of 11,303 instances, which to a certain extent causes the data sparseness. Therefore, these labels are deleted, and the remaining labels form label set L3, which contains 153 class labels. The experimental results are shown in Table 2. It can be seen that MLkNN and BP-MLL still have the best performance in each of the evaluation indicators, and the AP of BP-MLL has increased by nearly 3 percent.

We try to further reduce the sparseness of data and the labels whose frequencies are less than 10 by deleting them from the label set. The remained labels form the label set *L*_4_, which contains 71 class labels. The experimental results are shown in Table 3. It can be seen that MLkNN and BP-MLL have still the best performance in each of the evaluation indicators, and AP of BP-MLL is as high as 0.7413 ± 0.0100 by using the word vector feature.

In general, with the decrease of the label set size, the results keep increasing. MLkNN and BP-MLL have the best performance in each of the indicators. Whether the size of the label set is 233,153 or 71, the experimental results using the word vector as a feature are all better than those using LDA topics. We may get some reasons from the working process of the LDA model and Skip-gram model. The word representations computed using the Skip-gram model are very interesting since the learned vectors explicitly encode many linguistic regularities and patterns, while LDA topic model is a bag-of-words model that may ignore the relationships between words.

### 5.3. Experiment Results on Different Number of Topics

**Figure 4:**
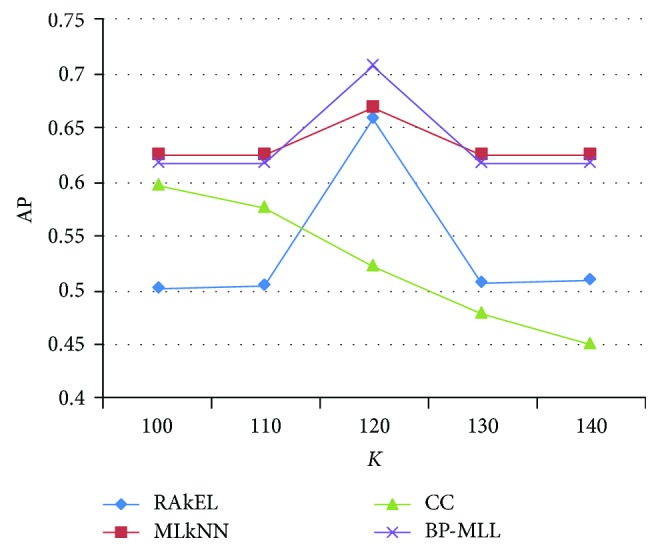
Experimental results on different number of topics.

As seen from the Section 4.1.1, the number of topics *K* must be given before the LDA model is trained. Since the number of topics selected in the above experiments is 120, *K* should be around 120 approximately. Thus, 100, 110, 130, and 140 are selected and they will be individually compared with *K* when it is 120. The purpose of this experiment is to study the effect of the topic number on the classification of the LDA. In the case of AP, the abscissa is the number of different topics, and the ordinate is the average precision of each method under different themes. It can be seen from Figure 4 that as the number of topics in the LDA continues to grow, the other three algorithms tend to be roughly the same. The exception is that the average precision of CC drops, reaching the highest point when the number of topics is approximately 120. The overall effects of MLkNN and BP-MLL are better than the other two algorithms. MLkNN is better than BP-MLL on both sides of the polyline, but in the middle part, BP-MLL is better than MLkNN.

### 5.4. Experiment Results on Different Number of Word Vector Dimensions

**Figure 5:**
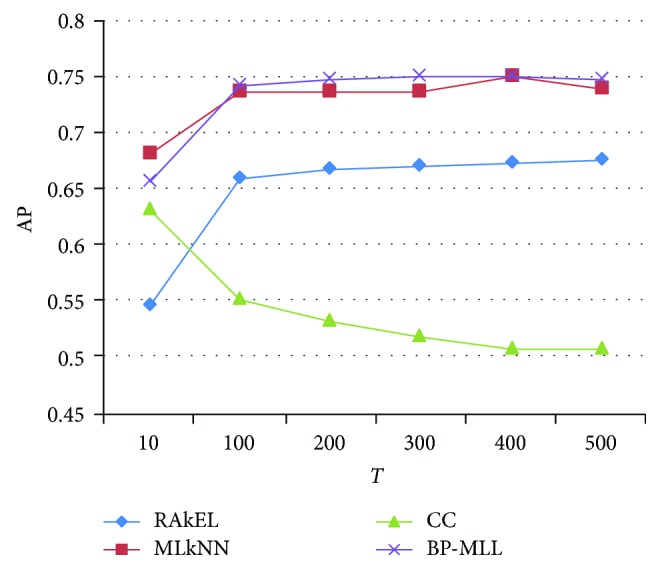
Experimental results on different word dimensions.

If the vector dimensions are not the same, it will affect the result. The different vector dimensions T of 10, 100, 200, 300, 400, and 500 are selected. The results are shown in Figure 5. In the case of AP, the abscissa is the word vector dimension, and the ordinate is the average precision of each method under different dimensions.

It can be seen from Figure 5 that as the vector dimension continues to grow, the AP of RAkEL, MLkNN, and BP-MLL tend to increase and the AP of CC drops. When the dimension is more than 100, the curve becomes gentle, but the time consumption will greatly increase. The overall effects of MLkNN and BP-MLL are better than the other two algorithms. MLkNN is better than BP-MLL on both sides of the polyline, but in the middle part, BP-MLL is better than MLkNN. Taking both the effectiveness and the efficiency into consideration, they are better when the vector dimension is 100.

## 6. Conclusion

In this paper, on the basis of the analysis of obstetric EMRs, the diagnosis assistant is regarded as a multilabel classification task. The LDA topic and the word vector trained by the Skip-gram model are adopted as the features and four methods; BP-MLL, RAkEL, MLkNN, and CC are utilized for multilabel classification. It also discusses the influence of the size of the label set, LDA topics, word vector dimensions and different, classifications on the experimental results. In general, the results using word vectors as features are slightly better than using LDA topics. The best result is achieved by BP-MLL with the word vector feature method. Its AP is up to 0.7413 ± 0.0100, when the label set size is 71 and the dimension of word vector is 100. The result of the diagnosis assistant can be introduced as a supplementary learning method for medical students. In this paper, the experiments are conducted on real cases of Chinese obstetric EMRs. The methods can be used for all kinds of medical records. Furthermore, the method proposed in this paper can be applied to English EMRs by treating the diagnosis assistant as multilabel classification.

From the discussion in this paper, the different features and classification methods in varying extent impact the experimental results. In the future work, we will focus more on mixing the extracted indicators with the help of the clinician to improve model performance. As for the multilabel classification, we will carry on the theoretical analysis of the performance differences between classifications and then propose the pertinent methods to get better results. It is expected that the result of the diagnosis assistant can provide an efficient assistant for the clinicians.

## Figures and Tables

**Table 1:** Results with |*L*_2_| = 233, *K* = 120, and *T* = 100.

Method	Feature	HL↓	C↓	OE↓	RL↓	AP↑
RAkEL	LDA	0.0085 ± 0.0002	124.5190 ± 2.9857	0.3479 ± 0.0192	0.2874 ± 0.0092	0.5727 ± 0.0090
	Word vector	0.0078 ± 0.0002	127.1671 ± 2.4166	0.2902 ± 0.0173	0.2984 ± 0.0104	0.5906 ± 0.0109

MLkNN	LDA	0.0078 ± 0.000	15.5416 ± 0.7277	0.2425 ± 0.0127	0.0292 ± 0.0009	0.6571 ± 0.0087
	Word vector	**0.0067 ± 0.0002**	13.6120 ± 0.6596	**0.2015 ± 0.0101**	0.0240 ± 0.0007	**0.7272 ± 0.0081**

CC	LDA	0.0093 ± 0.0002	109.6586 ± 2.9200	0.4908 ± 0.0150	0.2430 ± 0.0078	0.5073 ± 0.0097
	Word vector	0.0088 ± 0.0001	90.2732 ± 2.8796	0.4427 ± 0.0109	0.1960 ± 0.0070	0.5408 ± 0.0074

BP-MLL	LDA	0.0341 ± 0.0058	14.6960 ± 1.0139	0.2426 ± 0.0136	0.0276 ± 0.0020	0.6264 ± 0.0114
	Word vector	0.0244 ± 0.0012	**12.7561 ± 0.7484**	0.2431 ± 0.0136	**0.0225 ± 0.0009**	0.6588 ± 0.0091

**Table 2:** Results with |*L*_3_| = 153, *K* = 120, and *T* = 100.

Method	Feature	HL↓	C↓	OE↓	RL↓	AP↑
RAkEL	LDA	0.0120 ± 0.0003	67.4355 ± 1.5205	0.3044 ± 0.0125	0.2317 ± 0.0057	0.6205 ± 0.0074
	Word vector	0.0114 ± 0.0003	74.6069 ± 1.7410	0.2636 ± 0.0147	0.2527 ± 0.0088	0.6228 ± 0.0068

MLkNN	LDA	0.0113 ± 0.0002	11.3434 ± 0.5319	0.2511 ± 0.0074	0.0347 ± 0.0016	0.6650 ± 0.0065
	Word vector	**0.0101 ± 0.0002**	11.7522 ± 0.4557	**0.2015 ± 0.0101**	0.0318 ± 0.0015	**0.7289 ± 0.0086**

CC	LDA	0.0136 ± 0.0003	71.7498 ± 3.5310	0.4942 ± 0.0107	0.2533 ± 0.0142	0.5108 ± 0.0098
	Word vector	0.0134 ± 0.0002	61.0079 ± 1.8989	0.4427 ± 0.0109	0.2050 ± 0.0075	0.5430 ± 0.0075

BP-MLL	LDA	0.0362 ± 0.0043	10.2577 ± 0.5443	0.2531 ± 0.0087	0.0302 ± 0.0022	0.6522 ± 0.0149
	Word vector	0.0276 ± 0.0011	**10.6332 ± 0.4318**	0.2417 ± 0.0140	**0.0283 ± 0.0009**	0.6751 ± 0.0091

**Table 3:** Results with with |*L*_4_| = 71, *K* = 120, and *D* = 100.

Method	Feature	HL↓	C↓	OE↓	RL↓	AP↑
RAkEL	LDA	0.0244 ± 0.0004	26.3255 ± 1.1150	0.2799 ± 0.0123	0.1870 ± 0.0081	0.6575 ± 0.0090
	Word vector	0.0237 ± 0.0004	29.9007 ± 0.8173	0.2391 ± 0.0113	0.2074 ± 0.0071	0.6595 ± 0.0082

MLkNN	LDA	0.0241 ± 0.0003	9.2824 ± 0.2916	0.2498 ± 0.0112	0.0631 ± 0.0026	0.6697 ± 0.0085
	Word vector	**0.0214 ± 0.0005**	9.0997 ± 0.4973	**0.2014 ± 0.0103**	0.0547 ± 0.0033	0.7356 ± 0.0088

CC	LDA	0.0288 ± 0.0006	34.4526 ± 1.4447	0.4850 ± 0.0220	0.2729 ± 0.0130	0.5228 ± 0.0125
	Word vector	0.0285 ± 0.0004	30.4830 ± 0.7443	0.4427 ± 0.0109	0.2301 ± 0.0068	0.5509 ± 0.0069

BP-MLL	LDA	0.0458 ± 0.0046	7.4636 ± 0.4216	0.2521 ± 0.0128	0.0462 ± 0.0030	0.7081 ± 0.0098
	Word vector	0.0349 ± 0.0014	**7.4289 ± 0.4688**	0.2325 ± 0.0131	**0.0413 ± 0.0028**	**0.7413 ± 0.0100**
